# Relationship of pulmonary artery size and venovenous collaterals during staged single ventricle reconstruction and their impact on outcomes after Fontan procedure

**DOI:** 10.1093/icvts/ivaf070

**Published:** 2025-03-14

**Authors:** Teresa Lemmen, Thibault Schaeffer, Takuya Osawa, Carolin Niedermaier, Jonas Palm, Nicole Piber, Muneaki Matsubara, Paul Philipp Heinisch, Stanimir Georgiev, Alfred Hager, Peter Ewert, Jürgen Hörer, Masamichi Ono

**Affiliations:** Department of Congenital and Pediatric Heart Surgery, German Heart Center Munich, University Hospital of Technische Universität München, Munich, Germany; Division of Congenital and Pediatric Heart Surgery, University Hospital of Munich, Ludwig-Maximilians-Universität München, Munich, Germany; Europäisches Kinderherzzentrum München, Munich, Germany; Department of Congenital and Pediatric Heart Surgery, German Heart Center Munich, University Hospital of Technische Universität München, Munich, Germany; Division of Congenital and Pediatric Heart Surgery, University Hospital of Munich, Ludwig-Maximilians-Universität München, Munich, Germany; Europäisches Kinderherzzentrum München, Munich, Germany; Department of Congenital and Pediatric Heart Surgery, German Heart Center Munich, University Hospital of Technische Universität München, Munich, Germany; Division of Congenital and Pediatric Heart Surgery, University Hospital of Munich, Ludwig-Maximilians-Universität München, Munich, Germany; Europäisches Kinderherzzentrum München, Munich, Germany; Department of Congenital and Pediatric Heart Surgery, German Heart Center Munich, University Hospital of Technische Universität München, Munich, Germany; Division of Congenital and Pediatric Heart Surgery, University Hospital of Munich, Ludwig-Maximilians-Universität München, Munich, Germany; Europäisches Kinderherzzentrum München, Munich, Germany; Department of Congenital Heart Disease and Pediatric Cardiology, German Heart Center Munich, University Hospital of Technische Universität München, Munich, Germany; Department of Cardiovascular Surgery, German Heart Center Munich, University Hospital of Technische Universität München, Munich, Germany; Department of Congenital and Pediatric Heart Surgery, German Heart Center Munich, University Hospital of Technische Universität München, Munich, Germany; Division of Congenital and Pediatric Heart Surgery, University Hospital of Munich, Ludwig-Maximilians-Universität München, Munich, Germany; Europäisches Kinderherzzentrum München, Munich, Germany; Department of Congenital and Pediatric Heart Surgery, German Heart Center Munich, University Hospital of Technische Universität München, Munich, Germany; Division of Congenital and Pediatric Heart Surgery, University Hospital of Munich, Ludwig-Maximilians-Universität München, Munich, Germany; Europäisches Kinderherzzentrum München, Munich, Germany; Department of Congenital Heart Disease and Pediatric Cardiology, German Heart Center Munich, University Hospital of Technische Universität München, Munich, Germany; Department of Congenital Heart Disease and Pediatric Cardiology, German Heart Center Munich, University Hospital of Technische Universität München, Munich, Germany; Department of Congenital Heart Disease and Pediatric Cardiology, German Heart Center Munich, University Hospital of Technische Universität München, Munich, Germany; Department of Congenital and Pediatric Heart Surgery, German Heart Center Munich, University Hospital of Technische Universität München, Munich, Germany; Division of Congenital and Pediatric Heart Surgery, University Hospital of Munich, Ludwig-Maximilians-Universität München, Munich, Germany; Europäisches Kinderherzzentrum München, Munich, Germany; Department of Congenital and Pediatric Heart Surgery, German Heart Center Munich, University Hospital of Technische Universität München, Munich, Germany; Division of Congenital and Pediatric Heart Surgery, University Hospital of Munich, Ludwig-Maximilians-Universität München, Munich, Germany; Europäisches Kinderherzzentrum München, Munich, Germany

**Keywords:** single ventricle, pulmonary artery size, venovenous collaterals, bidirectional cavopulmonary shunt, total cavopulmonary connection

## Abstract

**OBJECTIVES:**

This study aimed to evaluate the relationship between pulmonary artery size and venovenous collaterals (VVCs) during staged single ventricle reconstruction.

**METHODS:**

Patients who underwent staged Fontan palliation between 2003 and 2023 were reviewed. The relationship between the pulmonary artery index and the development of VVCs was determined. Furthermore, the impact of pulmonary artery index and VVCs on in-hospital morbidities after the Fontan procedure was evaluated.

**RESULTS:**

A total of 377 patients were included. Median age at bidirectional cavopulmonary shunt (BCPS) and total cavopulmonary connection (TCPC) were 4.2 (3.3–6.2) months and 2.1 (1.7–2.6) years, respectively. VVCs were observed in 51 (13.5%) of the patients. Patients who developed VVCs showed higher pulmonary artery pressure (*P* = 0.024), higher transpulmonary gradient (*P* = 0.042), lower pulmonary artery index (*P* = 0.016) and lower right pulmonary artery index (*P* = 0.011) at the time of BCPS, compared to those without. However, the pulmonary artery index was similar in patients with and without VVCs at the time of TCPC. Higher transpulmonary gradient (*P* = 0.007) and lower pulmonary artery symmetry index (*P* = 0.032) at BCPS were identified as independent risks for developing VVCs. The existence of VVCs did not influence the postoperative course after TCPC. Notably, pulmonary artery symmetry index at BCPS was identified as an independent risk for prolonged pleural effusion (*P* = 0.018) and for chylothorax (*P* = 0.021).

**CONCLUSIONS:**

A small and unbalanced pulmonary artery at BCPS is associated with the postoperative development of VVCs.

## GRAPHICAL ABSTRACT

**Figure:**
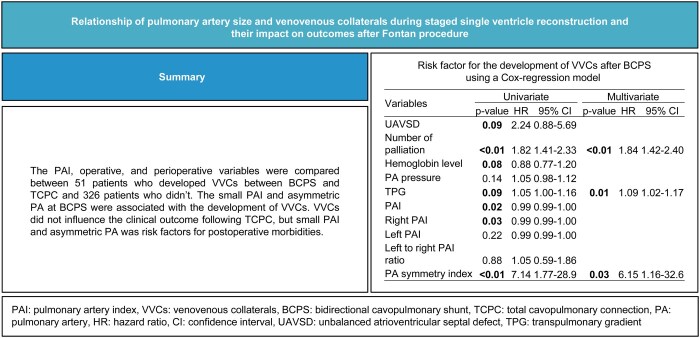

## INTRODUCTION

Total cavopulmonary connection (TCPC) has become the established palliative approach for patients with a functional single ventricle [1, 2]. It follows the bidirectional cavopulmonary shunt (BCPS), which leads to low-pressure pulmonary blood flow, reduces ventricular volume load and improves the likelihood of a successful TCPC [3, 4]. While BCPS provides haemodynamic benefits, a significant challenge is its complete reliance on passive venous flow through the superior vena cava (SVC) to maintain pulmonary circulation. The formation of venovenous collaterals (VVCs) from the upper systemic venous system to the lower systemic or pulmonary venous system could stem from increased SVC pressure [5–7]. Therefore, a small pulmonary artery (PA) size and the resulting reduced pulmonary artery blood flow could cause the development of VVCs and lead to insufficient growth of the central PA. Recent studies show that a small PA size is associated with a worse clinical status in patients after the Fontan procedure [8–10]. VVCs and aortopulmonary collaterals (APCs) develop under different circumstances. While APCs mainly develop to compensate for insufficient pulmonary blood flow, VVC formation is driven by elevated upper systemic venous pressure and the following desaturation and insufficient development of the central PA [7, 8]. Catheter interventions could become necessary as these collaterals might grow over time in proportion to central venous pressure [8–10]. To prevent those future complications, it is vital to understand whether a small PA size influences VVCs. However, few studies addressed the interaction of PA size and the development of VVCs.

In this study, we sought to determine the relation between PA size and the development of VVCs during staged single ventricle palliation between BCPS and TCPC. Furthermore, this study aimed to evaluate the effects of VVCs and PA size on the outcomes after TCPC. Outcomes of interest were whether small PA size influences the development of VVCs and whether small PA size and the presence of VVCs influence the in-hospital morbidities following TCPC.

## METHODS

### Ethical statement

This study was approved by the Institutional Review Board of the Technical University of Munich (approved number 2024–334-S-CB on the 8th of July 2024). Because of the retrospective nature of the study, the need for individual patient consent was waived.

### Patients and data collection

We reviewed all patients who underwent staged Fontan palliation from 2003 to 2023, and patients whose pre-BCPS and pre-TCPC angiograms were available were included. Baseline morphology, demographics and pre-, intra- and postoperative data were obtained through digital and paper chart reviews from each patient. Most follow-up data were retrieved from the institution’s regularly monitored single ventricle database.

### Surgical strategy and operative techniques

BCPS was conducted with standard cardiopulmonary bypass, and ante-grade pulmonary blood flow was eliminated in most cases [11]. The operative technique for TCPC was extracardiac TCPC [12].

### Cardiac catheterization, angiographic detection of VVCs and measurement of PA size

Prior to both BCPS and TCPC, all patients underwent cardiac catheterization. Cardiac catheterization during the interstage between BCPS and TCPC was indicated when patients demonstrated clinical symptoms such as desaturation/hypoxemia or haemodynamic instability. Haemodynamic measurements of pulmonary artery pressure (PAP) and left atrial pressure (LAP) were performed, and transpulmonary gradient (TPG) was calculated. Other parameters, including systemic ventricular systolic pressure (SVP) and end-diastolic pressure (EDP), mean arterial pressure and arterial oxygen saturation (SaO_2_), were measured. Conventional angiography was used to detect VVCs and other collaterals. Their presence was determined through angiograms of upper systemic veins, thoracic inferior vena cava and PAs [13, 14]. Technical details were described in our previous study [14]. According to Nakata *et al.*, the PA index was calculated using PA angiography [15]. The right and left PA indices were calculated by dividing the cross-sectional area of each PA branch by the body surface area. The symmetry index was calculated as described by Glatz *et al.* to evaluate the symmetric PA development [16]. The ratio of the left to the right PA index was calculated.

### Statistical analysis

Categorical variables are reported as absolute numbers and percentages. A chi-squared test was used to compare categorical data. Continuous variables are presented as medians with interquartile ranges (IQR). Normally distributed variables were compared using an independent sample t-test; for variables that were not normally distributed, a Mann–Whitney *U*-test was used. A Cox regression model was utilized to identify factors linked to the development of VVCs following BCPS. Variables reaching a significance level of <0.1 in the univariate analysis were included in the Cox multivariable models. Hazard ratios (HR) were calculated with 95% confidence intervals. The proportional hazard assumption was assessed using Schoenfeld residuals. A logistic regression analysis identified factors associated with morbidities following TCPC, with odds ratios (OR) calculated with 95% confidence intervals. The Kaplan–Meier method was used to estimate transplant-free survival after TCPC, with a log-rank test comparing patients with and without VVCs. Multiple imputation using predictive mean matching was used to find missing data and draw the regression models (‘mice’ package for R). Data analysis and graphing were carried out using the Statistical Package for the Social Sciences (SPSS) version 28.0 for Windows (IBM, Ehningen, Germany) and R-statistical software (R Foundation for Statistical Computing, Vienna, Austria).

## RESULTS

### Patient characteristics and perioperative data

**Figure 1:**
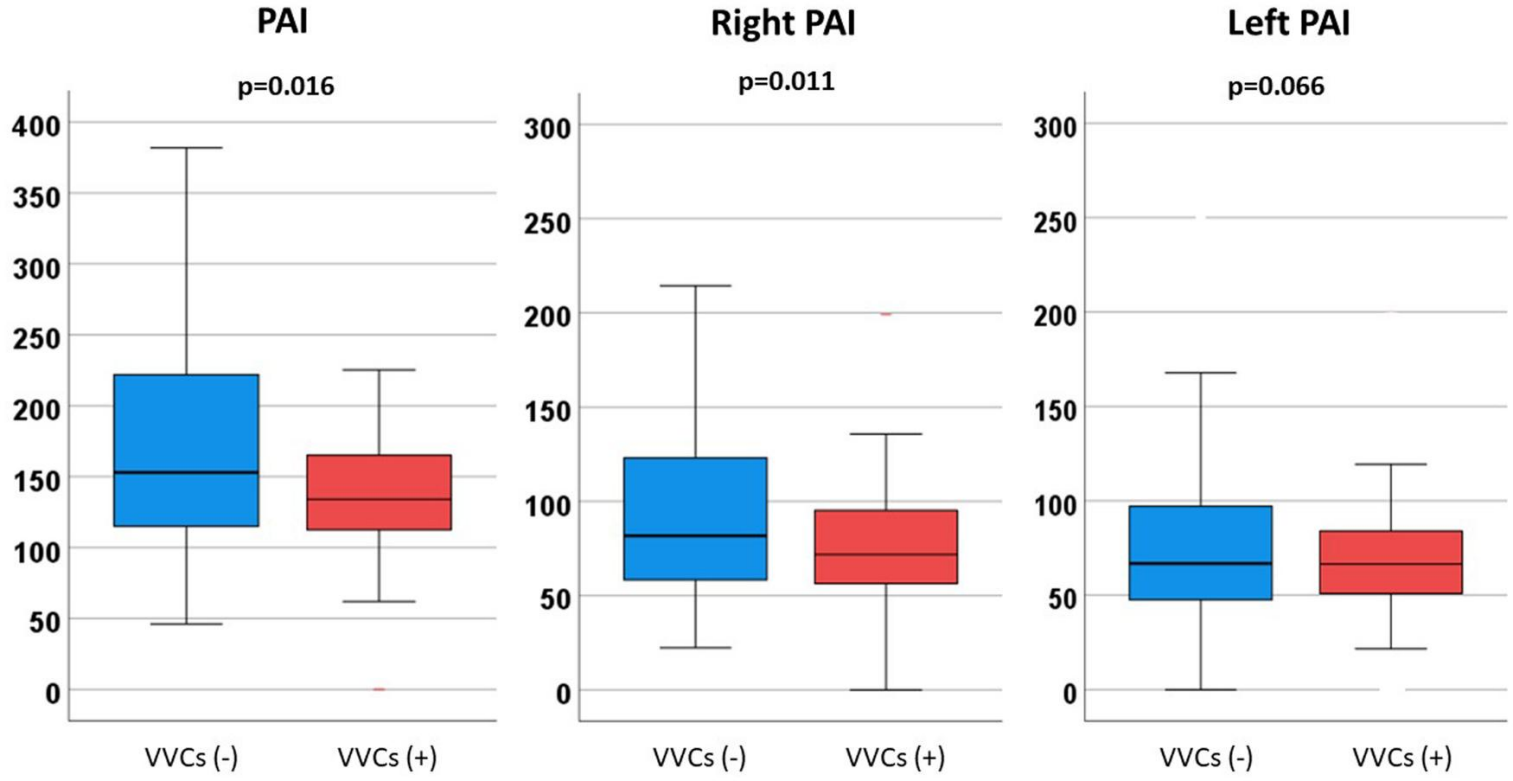
Box-and-whiskers plots showing PA index, right PA index and left PA index in patients with and without VVCs at the time of BCPS. PA: pulmonary artery, BCPS: bidirectional cavopulmonary shunt

**Figure 2:**
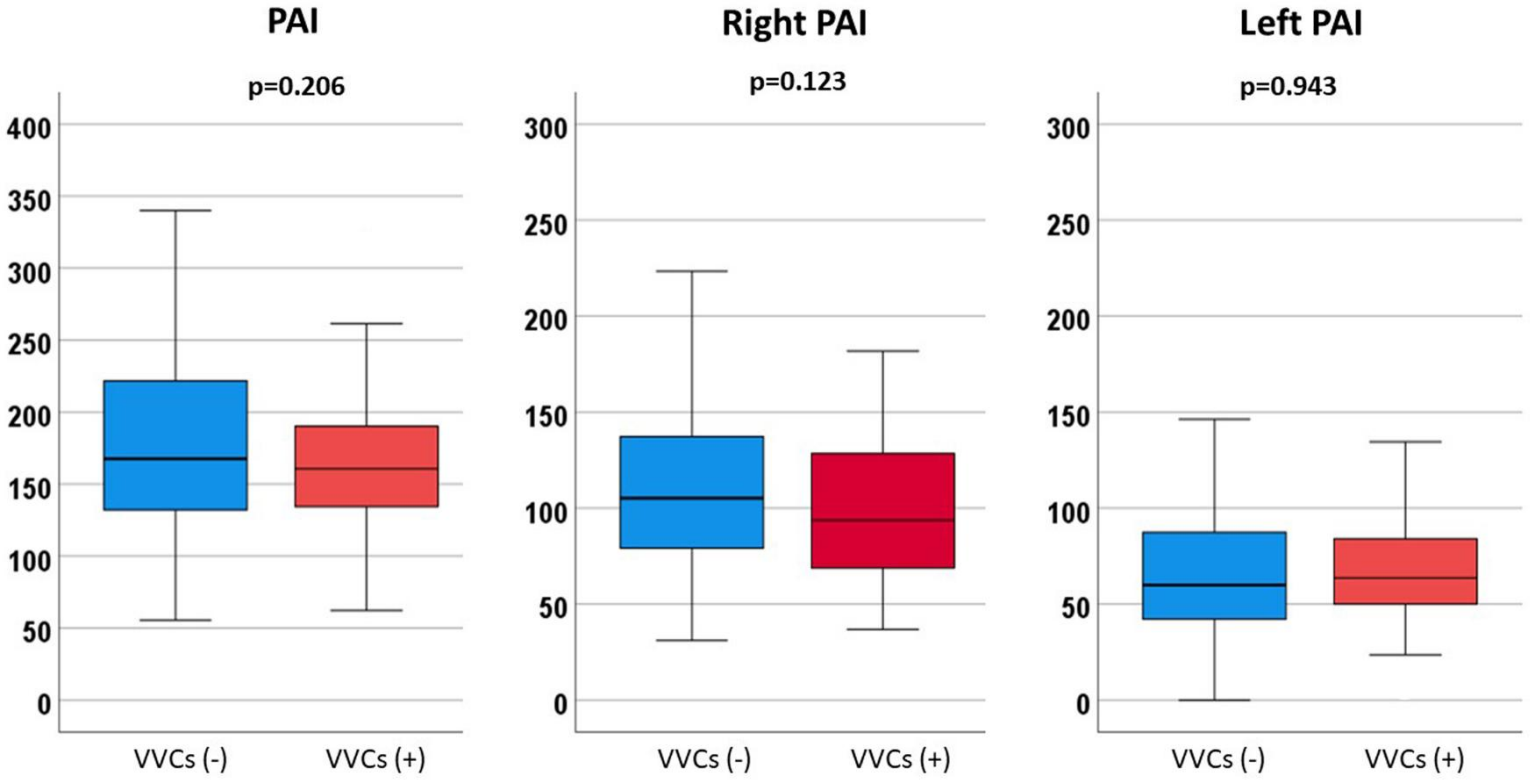
Box-and-whiskers plots showing PA index, right PA index and left PA index in patients with and without VVCs at the time of TCPC. PA: pulmonary artery, BCPS: bidirectional cavopulmonary shunt

A total of 377 patients were included in this study. VVCs between BCPS and TCPC were observed in 51 (13.5%) patients at a median of 34 (18–557) days after BCPS. Patients’ characteristics are presented in Table 1. Cardiac catheterization data are shown in Table 2. At the time of BCPS, median PAP (16 vs 14 mmHg, *P* = 0.024) and median TPG (8 vs 7 mmHg, *P* = 0.042) were higher in patients who developed VVCs than those who did not. PA index (135 vs 152 mm^2^/m^2^, *P* = 0.016) and right PA index (72 vs 82 mm^2^/m^2^, *P* = 0.011) were lower in patients with VVCs than those without (Fig. 1). Symmetry index (0.77 vs 0.71, *P* = 0.035) was higher in patients with VVCs than those without VVCs. At the time of TCPC, median PAP (11 vs 9 mmHg, *P* < 0.001) and median LAP (7 vs 5 mmHg, *P* < 0.001) were higher, and SaO_2_ (82 vs 83%, *P* = 0.033) was lower in patients with VVCs than those without. PA indexes at TCPC were similar between the groups (Fig. 2). During the interstage period between BCPS and TCPC, catheter interventions for PA were performed in 51 (13.5%) patients (17 (33.3%) patients with VVCs and 34 (10.4%) without VVCs). Surgery for PA was performed in 7 (1.9%) patients (3 (5.9%) with VVCs and 4 (1.2%) without VVCs). In 28 (7.4%) patients, VVCs were closed through an interventional procedure.

**Table 1:** Baseline cohort characteristics in patients with and without VVCs before TCPC

Variables: *N* (%) or median (IQR)	Total cases	VVCs (+)	VVCs (−)
Number of patients	377	51 (13.5)	326 (86.5)
Age at TCPC (years)	2.1 (1.7-2.6)	2.2 (1.8-2.7)	2.1 (1.7-2.5)
Weight at TCPC (kg)	11.5 (10.6-13.0)	11.2 (10.5-13.1)	11.5 (10.6-12.9)
Primary diagnosis			
HLHS	145 (38.5)	23 (45.1)	122 (37.4)
UVH	55 (14.6)	9 (17.6)	46 (14.1)
TA	52 (13.8)	5 (9.8)	47 (14.4)
DILV	45 (11.9)	3 (5.9)	42 (12.9)
PAIVS	20 (5.3)	3 (5.9)	17 (5.2)
ccTGA	19 (5.0)	1 (2.0)	18 (5.5)
UAVSD	18 (4.8)	5 (9.8)	13 (4.0)
Others	24 (6.4)	3 (5.9)	21 (6.4)
Dominant right ventricle	227 (60.2)	36 (70.6)	191 (58.6)
Associated cardiac anomaly			
TGA	76 (20.2)	9 (17.6)	67 (20.6)
DORV	42 (11.1)	7 (13.7)	35 (10.7)
CoA	39 (10.3)	4 (7.8)	35 (10.7)
Dextrocardia/Situs inversus	30 (8.0)	2 (3.9)	28 (8.6)
Heterotaxy	26 (6.9)	3 (5.9)	23 (7.1)
TAPVC/PAPVC	26 (6.9)	2 (3.9)	24 (7.4)
Systemic venous return anomaly	36 (9.5)	4 (7.8)	32 (9.8)
Palliation and pre-Fontan condition			
Norwood type procedure	203 (53.8)	30 (58.8)	173 (53.1)
Aortopulmonary shunt	74 (19.6)	11 (21.6)	63 (19.3)
Pulmonary artery banding	31 (8.2)	7 (13.7)	24 (7.4)
Palliative surgeries ≥3	42 (11.1)	12 (23.5)	30 (9.2)
BCPS			
Age at BCPS (months)	4.2 (3.3-6.2)	4.1 (3.2-5.8)	4.2 (3.3-6.2)
Weight at BCPS (kg)	5.3 (4.6-6.1)	4.9 (4.4-5.7)	5.4 (4.7-6.2)
Concomitant PA reconstruction	104 (27.6)	15 (29.4)	89 (27.3)
Bilateral BCPS	31 (8.2)	2 (3.9)	29 (8.9)
Additional APBF	13 (3.4)	4 (7.8)	9 (2.8)
Kawashima (Azygos cont.)	10 (2.7)	3 (5.9)	7 (2.1)

TCPC: total cavopulmonary connection; HLHS: hypoplastic left heart syndrome; UVH: univentricular heart; TA: tricuspid atresia; DILV: double inlet left ventricle; ccTGA: congenitally corrected TGA; PAIVS: pulmonary atresia and intact ventricular septum; UAVSD: unbalanced atrioventricular septal defect; TGA: transposition of the great arteries; DORV: double outlet right ventricle; CoA: coarctation of the aorta; T(P)APVC: Total (partial)anomalous pulmonary venous connection; BCPS: bidirectional cavopulmonary shunt; APBF: ante-grade pulmonary blood flow; PA: pulmonary artery.

**Table 2:** Pre-BCPS and pre-TCPC haemodynamic data and PAI

Variables: *N* (%) or median (IQR)	VVCs (+)	VVCs (−)	*P*-value
Number of patients	51 (13.5)	326 (86.5)	
Pre-BCPS			
Haemoglobin (g/dl)	13.2 (12.0-15.2)	14.1 (12.6-15.4)	0.053
Mean pulmonary artery pressure (mmHg)	16 (13-19)	14 (11-17)	**0.024**
Mean left atrium pressure (mmHg)	6 (6-8)	6 (5-8)	0.934
Transpulmonary gradient (mmHg)	8 (6-11)	7 (5-9)	**0.042**
Systolic ventricular pressure (mmHg)	79 (71-88)	79 (72-86)	0.991
Ventricular endo diastolic pressure (mmHg)	10 (8-11)	9 (8-11)	0.537
Aortic systolic pressure (mmHg)	78 (66-84)	78 (70-85)	0.976
Aortic mean pressure (mmHg)	48 (43-53)	49 (45-54)	0.351
Aortic oxygen saturation (%)	77 (71-79)	76 (72-80)	0.664
PAI	135 (113-175)	152 (115-222)	**0.016**
Right PAI	72 (56-96)	82 (58-123)	**0.011**
Left PAI	67 (51-85)	67 (47-97)	0.066
Left to right PAI ratio	0.88 (0.69-1.04)	0.85 (0.55-1.08)	0.935
Symmetry index	0.77 (0.63-0.89)	0.71 (0.51-0.88)	**0.035**
Pre-TCPC			
Haemoglobin (g/dl)	14.7 (13.8-16.7)	15.6 (14.6-16.7)	0.110
Mean pulmonary artery pressure (mmHg)	11 (9-12)	9 (8-11)	**<0.001**
Mean left atrium pressure (mmHg)	7 (5-8)	5 (4-7)	**<0.001**
Transpulmonary gradient (mmHg)	4 (3-5)	4 (3-5)	0.307
Systolic ventricular pressure (mmHg)	85 (80-95)	84 (77-92)	0.191
Ventricular end diastolic pressure (mmHg)	9 (8-10)	8 (8-9)	0.052
Aortic systolic pressure (mmHg)	85 (79-94)	82 (73-89)	0.967
Aortic mean pressure (mmHg)	59 (54-65)	58 (51-63)	0.351
Aortic oxygen saturation (%)	82 (77-85)	83 (80-86)	**0.033**
PAI	161 (131-193)	168 (132-222)	0.206
Right PAI	94 (68-131)	105 (79-137)	0.123
Left PAI	64 (49-85)	60 (42-88)	0.943
Left to right PAI ratio	0.66 (0.50-1.05)	0.58 (0.39-0.84)	0.122
Symmetry index	0.63 (0.49-0.79)	0.57 (0.39-0.77)	0.197

Data are presented with Median and IQR. BCPS: bidirectional cavopulmonary shunt; TCPC: total cavopulmonary connection. Bold values indicate p<0.05.

### Operative and postoperative data after TCPC

Perioperative data are shown in Table 3. Operative and postoperative variables were similar between the patients with and without VVCs. Transplant-free survival after TCPC was similar between the groups (89.9 vs 97.8% at 10 years, *P* = 0.302, Fig. S1). Post-TCPC catheter interventions for PA were performed in 79 (21.0%) patients (20 (39.2%) patients with VVCs and 59 (18.1%) without VVCs, *P* < 0.001). No patients had surgery for PA after TCPC. In 28 (7.4%) patients, VVCs were closed through an interventional procedure.

**Table 3:** Perioperative variables

Variables	Total	VVCs (+)	VVCs (−)	*P*-value
	377	51 (13.5)	326 (86.5)	
**Operative data**				
Extracardiac TCPC	377 (100.0)	51 (13.5)	326 (86.5)	
Conduit diameter (mm)				
16	4 (1.1)	2 (3.9)	2 (0.6)	
18	356 (94.4)	45 (88.2)	311 (95.4)	
20	17 (4.5)	4 (7.8)	13 (4.0)	
CPB time (min)	64 (47-89)	69 (53-89)	63 (46-88)	0.417
Aortic cross-clamp (AXC)	65 (17.2)	9 (17.6)	56 (17.2)	0.934
AXC time (min)	36 (22-56)	43 (25-61)	36 (19-57)	0.583
Fenestration at TCPC	28 (7.4)	4 (7.8)	24 (7.4)	0.918
Concomitant procedure	70 (18.6)	8 (15.7)	62 (19.0)	0.569
DKS	8 (2.1)	1 (2.0)	7 (2.1)	0.932
AVV procedure	39 (10.3)	6 (11.8)	33 (10.1)	0.720
PA reconstruction	16 (4.2)	1 (2.0)	15 (4.6)	0.384
Atrioseptectomy	13 (3.4)	1 (2.0)	12 (3.7)	0.531
SAS/VSD enlargement	8 (2.1)	1 (2.0)	7 (2.1)	0.932
Pacemaker implant	7 (1.9)	0 (0.0)	7 (2.1)	0.291
**Postoperative data**				
30-day mortality	3 (0.8)	1 (2.0)	2 (0.6)	0.314
ICU stay (days)	5 (4-8)	5 (4-8)	5 (4-8)	0.914
Hospital stay (days)	19 (13-26)	21 (14-24)	18 (13-26)	0.880
Complications				
Pleural effusion >7 days	217 (57.9)	30 (58.8)	187 (57.7)	0.882
Chylothorax	93 (24.8)	12 (23.5)	81 (25.0)	0.821
Ascites	61 (16.3)	16 (31.4)	45 (13.9)	**0.002**

Variables were presented in N (%) or median (IQR). RV: right ventricle; LV: left ventricle; TCPC: total cavopulmonary connection; CPB: cardiopulmonary bypass; AXC: aortic cross-clamp; DKS: Dames–Kaye–Stansel anastomosis; AVV: atrioventricular valve; PA: pulmonary artery; SAS: subaortic stenosis; VSD: ventricular septal defect; ICU: intensive care unit. Bold values indicate p<0.05.

### Risk factor analysis

A total of 70 and 32 missing observations were imputed for the Cox regression and logistic regression model, respectively. Original results without imputation (i.e. using listwise deletion) are available as Tables S1 and S2. Table 4 shows the risk factors for developing VVCs. Pre-BCPS PA index (*P* = 0.019, HR: 0.996) and pre-BCPS right PA index (*P* = 0.028, HR: 0.993) were identified as risk factors by univariate analysis but not by multivariate analysis. The multivariate analysis identified the number of palliative procedures (*P* < 0.001, HR: 1.840), pre-BCPS higher TPG (*P* = 0.007, HR: 1.090) and pre-BCPS lower symmetry index (*P* = 0.032, HR: 6.150) as independent risk factors for the development of VVCs. The risk factors for the postoperative morbidities after TCPC were performed using pre-BCPS and pre-TCPC catheterization data (Table 5). The multivariate analysis revealed pre-BCPS PAP (*P* = 0.040, OR: 1.05) and pre-BCPS symmetry index (*P* = 0.018, OR: 3.64) as independent risk factors for prolonged pleural effusion (>7 days). Pre-BCPS PAP (*P* = 0.012, OR: 1.07) and pre-BCPS symmetry index (*P* = 0.021, OR: 4.25) are independent risk factors for chylothorax.

**Table 4:** Risk factor for the development of VVCs after BCPS using a Cox-regression model

	Univariate			Multivariate		
Variables	*P*-value	HR	95% CI	*P*-value	HR	95% CI
Primary diagnosis						
HLHS	0.223	1.414	0.810-2.470			
TA	0.542	0.749	0.297-1.893			
DILV	0.273	0.520	0.162-1.675			
PAIVS	0.992	1.006	0.310-3.261			
UAVSD	**0.090**	2.239	0.881-5.687			
Associated anomalies						
Dominant right ventricle	0.101	1.676	0.903-3.110			
Dextrocardia/Situs inversus	0.215	0.408	0.099-1.682			
Heterotaxy	0.480	0.654	0.202-2.121			
TAPVC/PAPVC	0.248	0.433	0.105-1.793			
Initial palliations						
Norwood type procedure	0.332	1.324	0.751-2.332			
Aortopulmonary shunt	0.534	0.815	0.428-1.553			
Pulmonary artery banding	0.118	1.899	0.851-4.237			
Number of palliation	**<0.001**	1.816	1.413-2.334	**<0.001**	1.840	1.420-2.400
Pre-BCPS catheterization						
Haemoglobin (g/dl)	**0.084**	0.880	0.770-1.020			
Pulmonary artery pressure (mmHg)	**0.141**	1.051	0.980-1.120			
Left atrial pressure (mmHg)	0.936	0.996	0.892-1.111			
Transpulmonary gradient (mmHg)	**0.097**	1.050	1.001-1.157	**0.007**	1.090	1.020-1.170
Systemic ventricular pressure (mmHg)	0.945	1.001	0.977-1.025			
Endo-diastolic pressure (mmHg)	0.576	1.029	0.932-1.135			
Mean arterial pressure (mmHg)	0.474	0.986	0.949-1.025			
Arterial oxygen saturation (%)	0.610	1.010	0.972-1.049			
PA index	**0.019**	0.996	0.992-1.000			
Right PA index	**0.028**	0.993	0.987-1.000			
Left PA index	0.216	0.995	0.987-1.003			
Left to right PA index ratio	0.878	1.046	0.588-1.861			
Symmetry index	**0.006**	7.140	1.770-28.87	**0.032**	6.150	1.160-32.63
BCPS variables						
Age at BCPS (months)	0.411	0.983	0.942-1.025			
Weight at BCPS (kg)	0.765	0.982	0.875-1.104			
Bilateral BCPS	0.220	0.412	0.100-1.699			
Additional APBF	0.162	2.102	0.742-5.953			
Kawashima (Azygos cont.)	0.237	1.928	0.596-6.238			

The bold values denote <0.1 in univariate analysis and <0.05 in multivariate analysis. T(P)APVC: Total (partial)anomalous pulmonary venous connection; BCPS: bidirectional cavopulmonary shunt; APBF: ante-grade pulmonary blood flow.

**Table 5:** Risk factor for postoperative morbidities after BCPS

	Univariate			Multivariate		
Variables	*P*-value	OR	95% CI	*P*-value	OR	95% CI
**Prolonged pleural effusion (>7 days)**						
Pre-BCPS catheterization						
Pulmonary artery pressure (mmHg	**0.074**	1.05	1.00-1.10	**0.040**	1.05	1.00-1.11
Transpulmonary gradient (mmHg)	0.351	1.03	0.97-1.08			
PA index	**0.006**	1.00	0.99-1.00			
Right PA index	**0.011**	1.00	0.99-1.00			
Left PA index	**0.047**	1.00	0.99-1.00			
Left to right PA index ratio	0.614	1.00	0.75-1.65			
Symmetry index	**0.010**	3.33	1.34-8.42	**0.018**	3.64	1.26-10.72
Pre-TCPC catheterization						
Pulmonary artery pressure (mmHg)	0.604	1.02	0.94-1.12			
Transpulmonary gradient (mmHg)	0.821	1.02	0.89-1.16			
PA index	**0.009**	1.00	0.99-1.00			
Right PA index	0.126	1.00	0.99-1.00			
Left PA index	**0.005**	0.99	0.99-1.00			
Left to right PA index ratio	0.669	0.90	0.56-1.46			
Symmetry index	0.100	0.48	0.20-1.15			
**Chylothorax**						
Pre-BCPS catheterization						
Pulmonary artery pressure (mmHg	0.021	1.07	1.01-1.13	**0.012**	1.07	1.02-1.14
Transpulmonary gradient (mmHg)	0.386	1.03	0.97-1.09			
PA index	**0.041**	1.00	0.99-1.00			
Right PA index	**0.015**	0.99	0.99-1.00			
Left PA index	0.451	1.00	0.99-1.00			
Left to right PA index ratio	0.103	1.43	0.93-2.18			
Symmetry index	**0.005**	4.95	1.66-15.59	**0.021**	4.25	1.27-15.28
Pre-TCPC catheterization						
Pulmonary artery pressure (mmHg)	0.232	1.06	0.96-1.17			
Transpulmonary gradient (mmHg)	0.501	0.95	0.81-1.10			
PA index	0.436	1.00	0.99-1.00			
Right PA index	0.953	1.00	1.00-1.00			
Left PA index	0.124	0.99	0.99-1.00			
Left to right PA index ratio	0.171	0.66	0.35-1.17			
Symmetry index	0.562	0.74	0.27-2.01			

The bold values denote significant in statistical analysis <0.05. T(P)APVC: total (partial)anomalous pulmonary venous connection; BCPS: bidirectional cavopulmonary shunt; APBF: ante-grade pulmonary blood flow.

## DISCUSSION

The development of VVCs after BCPS was associated with small PA indices and asymmetric pulmonary arteries at BCPS. Although the VVCs between BCPS and TCPC did not influence in-hospital morbidities after TCPC, small PA size and asymmetric pulmonary arteries at BCPS were a risk for prolonged pleural effusion and chylothorax following TCPC.

### Development of VVCs after BCPS and its relationship to PA size

Upper systemic venous hypertension, a consequence of BCPS physiology, may contribute to the formation of VVCs. In this study, pre-BCPS high TPG and PA symmetry indices were independent risks for the development of VVCs. McElhinney *et al.* demonstrated that TPG early after BCPS was higher in patients who developed VVCs [5]. Magee *et al.* identified increased TPG as a risk factor for VVCs [6]. However, these previous studies did not evaluate the PA size. To the best of our knowledge, our results are the first to demonstrate PA size as a risk for the development of VVCs.

At TCPC, patients who developed VVCs demonstrated higher PAP, higher LAP and lower SaO_2_. These findings suggest that despite VVCs, PAP was still higher in patients with VVCs than those without. Interestingly, patients with VVCs had comparable PA indices at TCPC. PA growth between BCPS and TCPC in patients with VVCs should be analyzed in future studies. In this study, the number of palliations was identified as a risk for the development of VVCs. Our previous study demonstrated that the number of palliations was a risk for the development of APCs [17]. Repeated surgical procedures might contribute to the accelerated development of both APCs and VVCs.

### Relationship between PA size, the development of VVCs and outcomes after TCPC

No previous study has demonstrated that VVCs impact mortality or morbidity after the Fontan procedure. Similarly, our results also showed no influence on survival after TCPC. VVCs after BCPS may function as natural shunts, lowering the SVC pressure and could either regress or disappear after TCPC. The effects of interventional closure of VVCs were also reported [18]. On the contrary, pre-BCPS PA indices were associated with postoperative prolonged pleural effusion and chylothorax. It is worth noting that pre-BCPS PA indices, not pre-TCPC PA indices, were identified as risks. Pre-BCPS PA indices were associated with the development of VVCs and postoperative morbidities after TCPC. To our knowledge, these findings are the first to be reported. Therefore, PA size at BCPS might be an important factor for the outcomes after staged Fontan palliation, although the reason why the PA size at BCPS is more important than the one at TCPC remains unclear. A recent study by Ridderbos *et al.* reported an association between small PA size after the Fontan procedure and worse clinical status [9]. Several reports suggested that a small pre-TCPC PA size does not impact outcomes after the Fontan procedure [19–21]. However, its influence remains widely debated. Meanwhile, Itatani *et al.* reported that a small PA size results in high energy loss in the TCPC pathway [22]. Recent studies and the results of this study re-emphasize the importance of PA size during the staged Fontan palliation. Further studies are mandatory to clarify the impact of PA size on long-term outcomes after TCPC in the current cohort of patients.

### Study limitations

This study was limited by its retrospective and single-centre study design. Surgical and medical management may have changed during the study period, probably influencing the long-term outcomes. The improvement of imaging technologies by angiogram in the recent era might also create a bias in the detection of VVCs. VVCs were detected by cardiac catheterization, and the flow of VVCs could not be quantified. Missing flow measurements of VVCs by cardiac magnetic resonance imaging is another limitation. The interaction of VVCs and APCs was not analyzed in this study. The objective of the study is to investigate whether PAI increases the risk of VCC. Therefore, the only relevant finding is the association between PAI and VVCs. The role of other covariates is marginally investigated.

## CONCLUSIONS

This study reveals a correlation between the PA index at BCPS and the occurrence of VVCs. The development of VVCs after BCPS did not influence clinical outcomes after TCPC, whereas small PA size and asymmetric pulmonary arteries at BCPS were a risk for postoperative prolonged pleural effusion and chylothorax following TCPC.

## SUPPLEMENTARY MATERIAL

Supplementary material is available at *ICVTS* online.

## FUNDING

This study was supported by grants from the Förderverein des Deutschen Herzzentrums München.

## CONFLICT OF INTEREST

The authors declare no potential conflicts of interest concerning the research, authorship, or publication of this article.

## Supplementary Material

ivaf070_Supplementary_Data

## Data Availability

The data are available from the corresponding author upon reasonable request.

## Notes

Presented at the 38th EACTS Annual Meeting, Lisbon, Portugal, October 9–12, 2024.

## ABBREVIATIONS

APCs: Aortopulmonary collaterals
BCPS: Bidirectional cavopulmonary shunt
EDP: End-diastolic pressure
HLHS: Hypoplastic left heart syndrome
HR: Hazard ratio
IQR: Interquartile ranges
LAP: Left atrial pressure
OR: Odds ratio
PAP: Pulmonary artery pressure
SaO_2_: Arterial oxygen saturation
SVP: Systemic ventricular pressure
TCPC: Total cavopulmonary connection
TPG: Transpulmonary gradient
VVCs: Venovenous collaterals
