# Supplementary material for: Relationship of pulmonary artery size and venovenous collaterals during staged single ventricle reconstruction and their impact on outcomes after Fontan procedure
Source: Interdiscip Cardiovasc Thorac Surg. 2025 Mar 14;40(3):ivaf070. doi: 10.1093/icvts/ivaf070 (PMC11951102; doi:10.1093/icvts/ivaf070)
Supplement: ivaf070_Supplementary_Data [file ivaf070_supplementary_data.zip › Supplementary Tables22022025.docx]

**Supplementary Tables**

**Supplementary Table S1**

| Table S1: Risk factors for the development of VVCs after BCPS using a Cox-regression model | | | | | | | |
| --- | --- | --- | --- | --- | --- | --- | --- |
|  | Univariate | | |  | Multivariate | | |
| Variables | p-value | HR | 95% CI |  | p-value | HR | 95% CI |
| Primary diagnosis |  |  |  |  |  |  |  |
| HLHS | 0.223 | 1.414 | 0.810-2.470 |  |  |  |  |
| TA | 0.542 | 0.749 | 0.297-1.893 |  |  |  |  |
| DILV | 0.273 | 0.520 | 0.162-1.675 |  |  |  |  |
| PAIVS | 0.992 | 1.006 | 0.310-3.261 |  |  |  |  |
| UAVSD | **0.090** | 2.239 | 0.881-5.687 |  |  |  |  |
| Associated anomalies |  |  |  |  |  |  |  |
| Dominant right ventricle | 0.101 | 1.676 | 0.903-3.110 |  |  |  |  |
| Dextrocardia/Situs Inversus | 0.215 | 0.408 | 0.099-1.682 |  |  |  |  |
| Heterotaxy | 0.480 | 0.654 | 0.202-2.121 |  |  |  |  |
| TAPVC/PAPVC | 0.248 | 0.433 | 0.105-1.793 |  |  |  |  |
| Initial palliations |  |  |  |  |  |  |  |
| Norwood type procedure | 0.332 | 1.324 | 0.751-2.332 |  |  |  |  |
| Aortopulmonary Shunt | 0.534 | 0.815 | 0.428-1.553 |  |  |  |  |
| Pulmonary artery banding | 0.118 | 1.899 | 0.851-4.237 |  |  |  |  |
| Number of palliations | **<0.001** | 1.816 | 1.413-2.334 |  | **0.002** | 3.069 | 1.516-6.212 |
| Pre-BCPS catheterization |  |  |  |  |  |  |  |
| Hemoglobin (g/dl) | **0.039** | 0.855 | 0.737-0.992 |  |  |  |  |
| Pulmonary artery pressure (mmHg | **0.031** | 1.081 | 1.007-1.161 |  |  |  |  |
| Left atrial pressure (mmHg) | 0.936 | 0.996 | 0.892-1.111 |  |  |  |  |
| Transpulmonary gradient (mmHg) | **0.046** | 1.076 | 1.001-1.157 |  | **0.007** | 1.332 | 1.083-1.640 |
| Systemic ventricular pressure (mmHg) | 0.945 | 1.001 | 0.977-1.025 |  |  |  |  |
| Endo-diastolic pressure (mmHg) | 0.576 | 1.029 | 0.932-1.135 |  |  |  |  |
| Mean arterial pressure (mmHg) | 0.474 | 0.986 | 0.949-1.025 |  |  |  |  |
| Arterial oxygen saturation (%) | 0.610 | 1.010 | 0.972-1.049 |  |  |  |  |
| PA index | **0.061** | 0.996 | 0.992-1.000 |  |  |  |  |
| Right PA index | **0.052** | 0.993 | 0.987-1.000 |  | **0.015** | 0.984 | 0.971-0.997 |
| Left PA index | 0.216 | 0.995 | 0.987-1.003 |  |  |  |  |
| Left to right PA index ratio | 0.878 | 1.046 | 0.588-1.861 |  |  |  |  |
| Symmetry index | **0.080** | 3.869 | 0.851-17.592 |  |  |  |  |
| BCPS variables |  |  |  |  |  |  |  |
| Age at BCPS (months) | 0.411 | 0.983 | 0.942-1.025 |  |  |  |  |
| Weight at BCPS (kg) | 0.765 | 0.982 | 0.875-1.104 |  |  |  |  |
| Bilateral BCPS | 0.220 | 0.412 | 0.100-1.699 |  |  |  |  |
| Additional APBF | 0.162 | 2.102 | 0.742-5.953 |  |  |  |  |
| Kawashima (Azygos cont.) | 0.237 | 1.928 | 0.596-6.238 |  |  |  |  |
| The bold values denote <0.1 in univariate analysis and <0.05 in multivariate analysis | | | | | | |  |
| T(P)APVC; Total (partial)anomalous pulmonary venous connection | | | |  |  |  |  |
| BCPS: bidirectional cavopulmonary shunt, APBF: ante-grade pulmonary blood flow | | | | | |  |  |

**Supplementary Table S2**

| Table S2: Risk factors for postoperative morbidities after TCPC | | | |  |  |  |  |
| --- | --- | --- | --- | --- | --- | --- | --- |
|  | Univariate | | |  | Multivariate | | |
| Variables | p-value | OR | 95% CI |  | p-value | OR | 95% CI |
| **Prolonged pleural effusion (>7 days)** | |  |  |  |  |  |  |
| Pre-BCPS catheterization |  |  |  |  |  |  |  |
| Pulmonary artery pressure (mmHg) | 0.368 | 1.027 | 0.969-1.090 |  |  |  |  |
| Transpulmonary gradient (mmHg) | 0.728 | 1.011 | 0.950-1.077 |  |  |  |  |
| PA index | **0.003** | 0.996 | 0.994-0.999 |  | **0.005** | 0.996 | 0.993-0.999 |
| Right PA index | **0.002** | 0.994 | 0.990-0.998 |  |  |  |  |
| Left PA index | **0.049** | 0.995 | 0.990-1.000 |  |  |  |  |
| Left to right PA index ratio | 0.341 | 1.245 | 0.793-1.954 |  |  |  |  |
| Symmetry index | **0.042** | 2.989 | 1.042-8.576 |  |  |  |  |
| Pre-TCPC catheterization |  |  |  |  |  |  |  |
| Pulmonary artery pressure (mmHg) | 0.491 | 1.031 | 0.945-1.126 |  |  |  |  |
| Transpulmonary gradient (mmHg) | 0.979 | 0.998 | 0.872-1.142 |  |  |  |  |
| PA index | **0.010** | 0.996 | 0.993-0.999 |  |  |  |  |
| Right PA index | 0.127 | 0.997 | 0.992-1.001 |  |  |  |  |
| Left PA index | **0.004** | 0.991 | 0.985-0.997 |  |  |  |  |
| Left to right PA index ratio | 0.363 | 0.790 | 0.475-1.313 |  |  |  |  |
| Symmetry index | 0.126 | 0.490 | 0.196-1.222 |  |  |  |  |
| **Chylothorax** |  |  |  |  |  |  |  |
| Pre-BCPS catheterization |  |  |  |  |  |  |  |
| Pulmonary artery pressure (mmHg | 0.232 | 1.041 | 0.975-1.111 |  |  |  |  |
| Transpulmonary gradient (mmHg) | 0.852 | 1.007 | 0.937-1.081 |  |  |  |  |
| PA index | **0.017** | 0.996 | 0.993-0.999 |  |  |  |  |
| Right PA index | **0.007** | 0.993 | 0.988-0.998 |  | **0.004** | 0.992 | 0.986-0.997 |
| Left PA index | 0.221 | 0.996 | 0.990-1.002 |  |  |  |  |
| Left to right PA index ratio | 0.067 | 1.538 | 0.971-2.435 |  |  |  |  |
| Symmetry index | **0.027** | 3.957 | 1.171-13.366 |  |  |  |  |
| Pre-TCPC catheterization |  |  |  |  |  |  |  |
| Pulmonary artery pressure (mmHg) | 0.192 | 1.068 | 0.968-1.179 |  |  |  |  |
| Transpulmonary gradient (mmHg) | 0.810 | 0.981 | 0.837-1.149 |  |  |  |  |
| PA index | 0.565 | 0.999 | 0.995-1.003 |  |  |  |  |
| Right PA index | 0.994 | 1.000 | 0.995-1.005 |  |  |  |  |
| Left PA index | 0.287 | 0.996 | 0.989-1.003 |  |  |  |  |
| Left to right PA index ratio | 0.332 | 0.737 | 0.398-1.365 |  |  |  |  |
| Symmetry index | 0.765 | 0.855 | 0.305-2.392 |  |  |  |  |
| The bold values denote significant in statistical analysis <0.05 | | | |  |  |  |  |
| T(P)APVC; Total (partial)anomalous pulmonary venous connection | | | |  |  |  |  |
| BCPS: bidirectional cavopulmonary shunt, APBF: ante-grade pulmonary blood flow | | | | | |  |  |
