# Supplementary figures and images for: Relationship of pulmonary artery size and venovenous collaterals during staged single ventricle reconstruction and their impact on outcomes after Fontan procedure
Source: Interdiscip Cardiovasc Thorac Surg. 2025 Mar 14;40(3):ivaf070. doi: 10.1093/icvts/ivaf070 (PMC11951102; doi:10.1093/icvts/ivaf070)

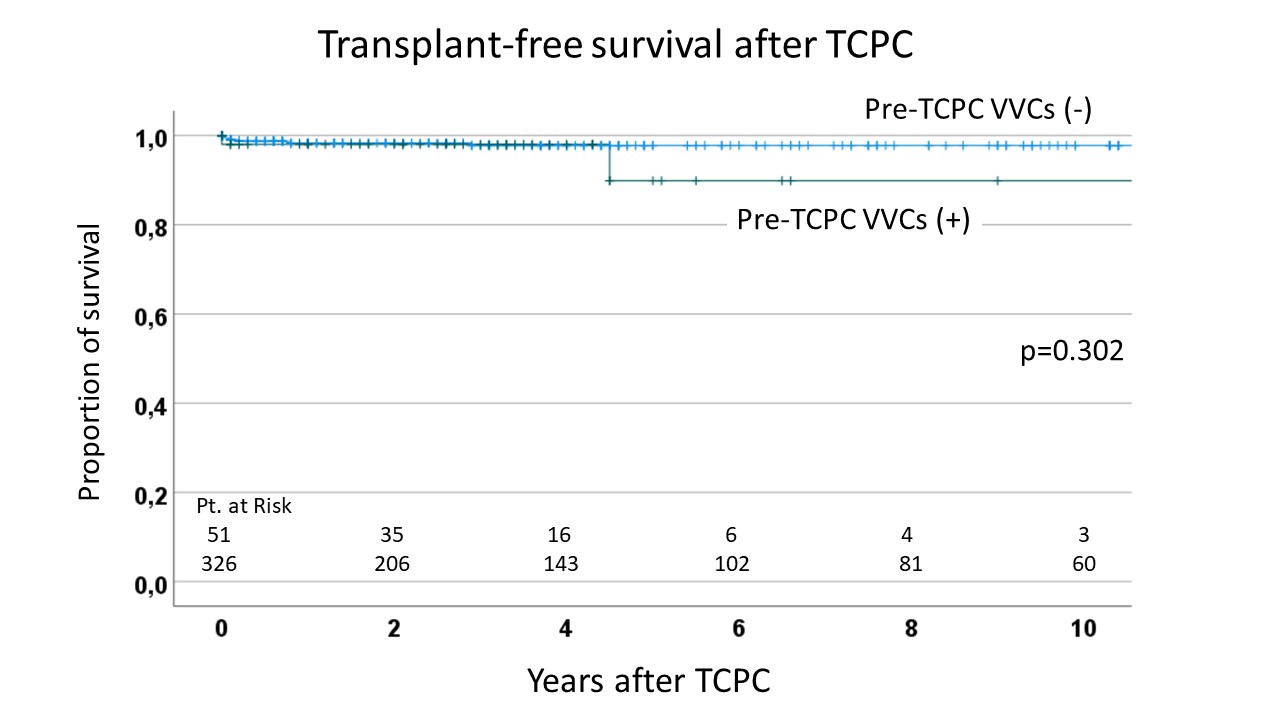

Supplement: ivaf070_Supplementary_Data [file ivaf070_supplementary_data.zip › ManuskriptSuppleFigure1KM_EJCTS_Teresa05072024.jpg]
